# Giant cell tumor of bone: exploring HtrA1 as a potential predictor of recurrence

**DOI:** 10.1007/s00402-026-06374-5

**Published:** 2026-06-12

**Authors:** Akif Mirioglu, Kaan Ali Dalkir, Mehmet Yigit Gokmen, Melih Bagir, Kivilcim Eren Ates, Mehmet Ali Deveci, Gülfiliz Gönlüşen, Hilmi Serdar Ozbarlas

**Affiliations:** 1 Department of Orthopaedics and Traumatology, Cukurova University, Adana, Turkey; 2 Department of Orthopaedics and Traumatology, Çanakkale Onsekiz Mart University, Çanakkale, Turkey; 3 Department of Pathology, Cukurova University, Adana, Turkey; 4 Department of Orthopaedics and Traumatology, Koç University, Istanbul, Turkey

**Keywords:** Giant cell tumor of bone, High-temperature requirement A serine peptidase 1, Immunohistochemistry, Prognosis

## Abstract

**Introduction:**

Giant cell tumor of bone (GCTB) is a locally aggressive tumor with a considerable risk of recurrence, but reliable predictive markers are limited. High-temperature requirement A serine peptidase 1 (HtrA1) has been implicated in the progression of several cancers and may also play a role in GCTB recurrence.

**Materials and methods:**

This retrospective study included 34 patients with GCTB treated with curettage between 2002 and 2016. HtrA1 expression was evaluated separately in giant cells (GC) and mononuclear cells (MNC) using immunohistochemistry. Based on high or low expression in each cell type, patients were categorized into four expression patterns: High GC / High MNC, High GC / Low MNC, Low GC / High MNC, and Low GC / Low MNC. Clinical data, including recurrence status and time to recurrence, were analyzed.

**Results:**

The High GC / Low MNC group showed the highest recurrence rate at 71.4%, compared with 28.6% in the High GC / High MNC group and 31.6% in the Low GC / Low MNC group. No recurrence was observed in the Low GC / High MNC group, which included only one patient. The High GC / Low MNC group showed a non-significant trend toward higher recurrence compared with all other groups combined, with a similar trend toward shorter recurrence-free survival.

**Conclusion:**

HtrA1 expression patterns in GCTB may provide preliminary insight into recurrence risk. Although no statistically significant association was demonstrated, the observed trends suggest potential prognostic relevance and warrant validation in larger cohorts.

## Introduction

 Giant cell tumor of bone (GCTB) is a locally aggressive tumor that presents significant challenges during the treatment, including recurrences and lung metastasis. Despite various advances in treatment strategies, the rates of these complications have remained consistent since the tumor was first described. Different strategies have been developed against both local recurrences and their metastatic potential [1, 2]. However, recurrence rates are still reported to be around 15–30%, with a metastasis rate of approximately 5% [3]. This can be attributed to an incomplete understanding of the nature of the disease; in other words, the secret of its biological behavior remains unresolved. Despite certain risk factors for recurrences and metastasis of disease have been described, these factors were generally based on retrospective patient characteristics and derived from the treatment outcomes [4, 5]. Both the microscopic and molecular features of the tumor have drawn significant attention from researchers, leading to the uncovering of versatile characteristics of this locally aggressive tumor. GCTB is mainly composed of three different types of cells: Monotonous sheets of round to oval or spindle-shaped cells, macrophage-like mononuclear cells, and osteoclastic giant cells [6]. The primary pathophysiology involves the increased expression of RANKL on the surface of the spindle-shaped tumoral cells, which binds to RANK receptors on the osteoclasts. This interaction overstimulates the NF-ĸB pathway, resulting in the formation of osteoclastic giant cells. Denosumab was developed to inhibit this pathway [7]. However, while Denosumab was shown to be effective in increasing sclerosis, the rising recurrence rates led to confusion and ultimately limited its use in treating local disease [8]. In 2013, a new mutation was identified in the mononuclear stromal component of GCTB, known as the H3.3 mutation, which is present in nearly 90% of tumors [9, 10]. Despite these advancements, the exact process that initiates tumor formation remains unclear.

The high-temperature requirement A (HtrA) serine proteases family primarily controls intracellular protein quality and has been defined as playing a role in arthritic and neurodegenerative diseases. Recently, these proteases have also gained popularity in cancer research fields. The expressions of HtrA1 and HtrA3 have been identified as acting like tumor suppressors and are downregulated in many cancers [11]. Osteoblasts and osteoclasts also secrete HtrA1, playing a role that cannot be ignored [12]. Therefore, it may also have a possible role in GCTB and deserves to be investigated. In this paper, we aim to show whether HtrA1 could help predict the outcome or clinical course of the patients with GCTB.

## Materials and methods

**Fig. 1:**
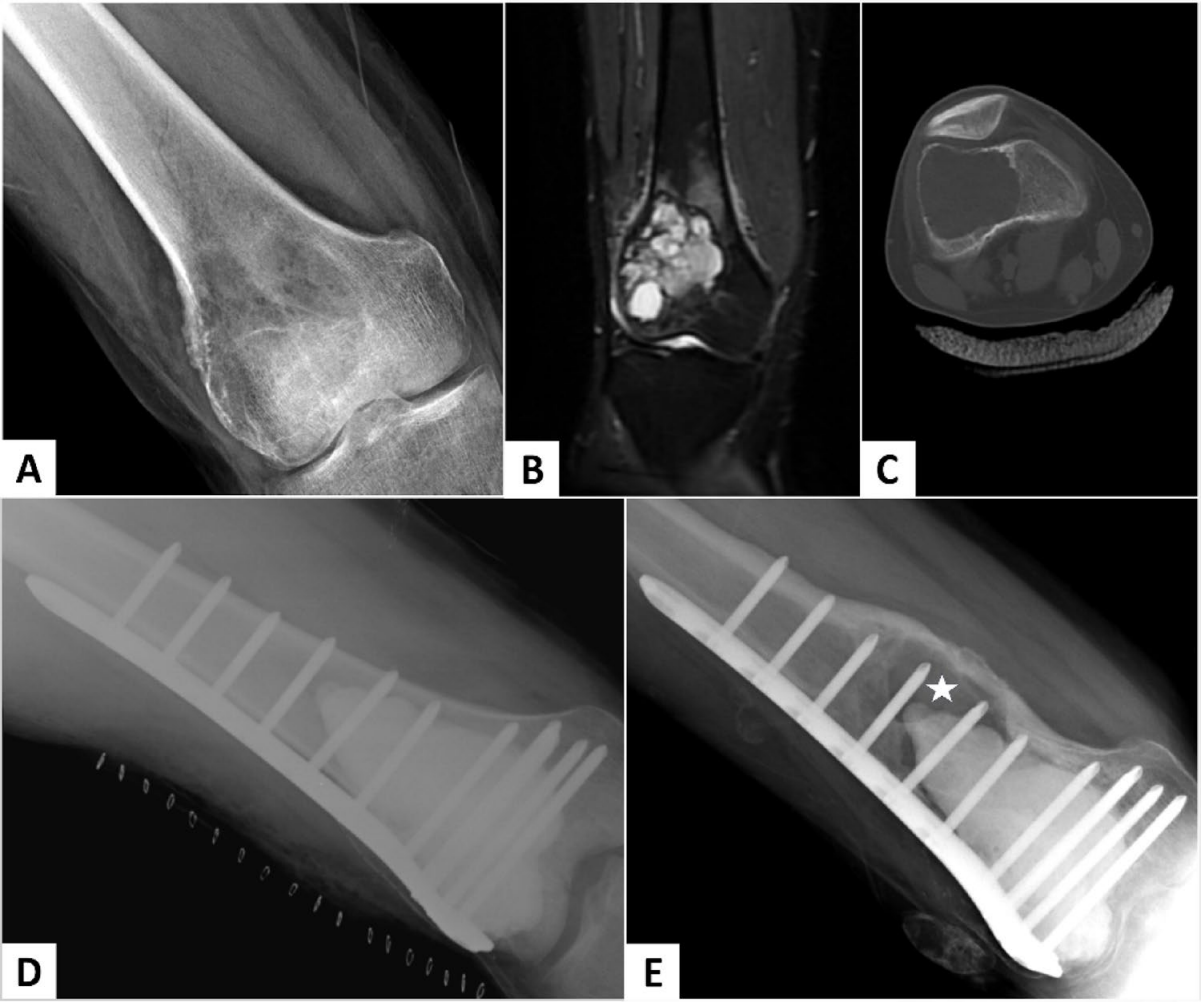
A 31-year-old patient with a giant cell tumor of the right distal femur. Preoperative lateral radiograph (**A**), coronal MRI (**B**), and axial CT image (**C**) show an expansile lytic lesion in the distal femur. Early postoperative radiograph after curettage, cementation, and plate fixation (**D**). Radiographic recurrence was observed around the cement mantle (asterisk) (**E**)

Ethics committee approval was obtained from the Non-Interventional Clinical Research Ethics Committee of Çukurova University with meeting number 101 and decision number 18. A total of 34 patients who were diagnosed and treated with GCTB between 2002 and 2016 were enrolled in the study. Patients were included if they had a histopathologically confirmed diagnosis of GCTB, underwent treatment at our institution, had available primary tumor pathology specimens for immunohistochemical evaluation, and had a minimum clinical follow-up period of 24 months for recurrence assessment. Patients were excluded if their follow-up period was less than 24 months, received neoadjuvant denosumab, or they underwent surgery at another institute without providing the specimen. For each patient, comprehensive clinical and pathological data were obtained from the hospital database. This included demographics (age, gender), tumor location, treatment modality, as well as follow-up information regarding recurrence. Time to recurrence was defined as the interval between the index surgery and documented local recurrence. Campanacci grading was assessed on preoperative radiographs by two orthopedic surgeons experienced in musculoskeletal oncology, and the final grade was determined by consensus. All patients underwent intralesional curettage followed by high-speed burring. After curettage, the defect was reconstructed with either polymethylmethacrylate cement or bone graft. Both the reconstruction method and the use of local adjuvants were recorded for each patient because of their potential influence on local recurrence. Local adjuvants included alcohol, phenol, and hydrogen peroxide, either alone or in combination (Fig. 1).

**Fig. 2:**
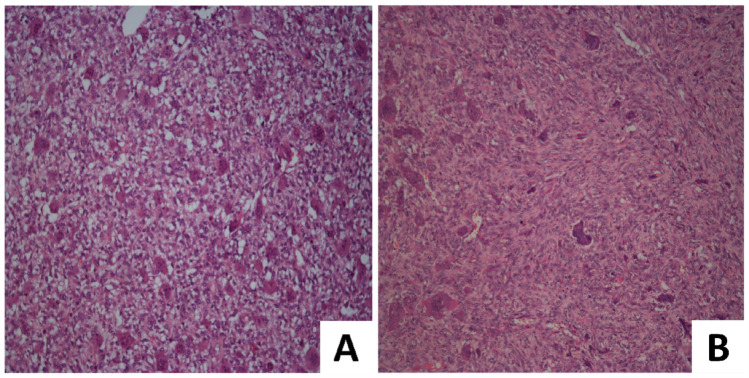
Hematoxylin & Eosin (X100): Mononuclear cells and homogeneously distributed giant cells in conventional GCT (**A**). Spindle-shaped mononuclear cells observed in GCT (**B**)

**Fig. 3:**
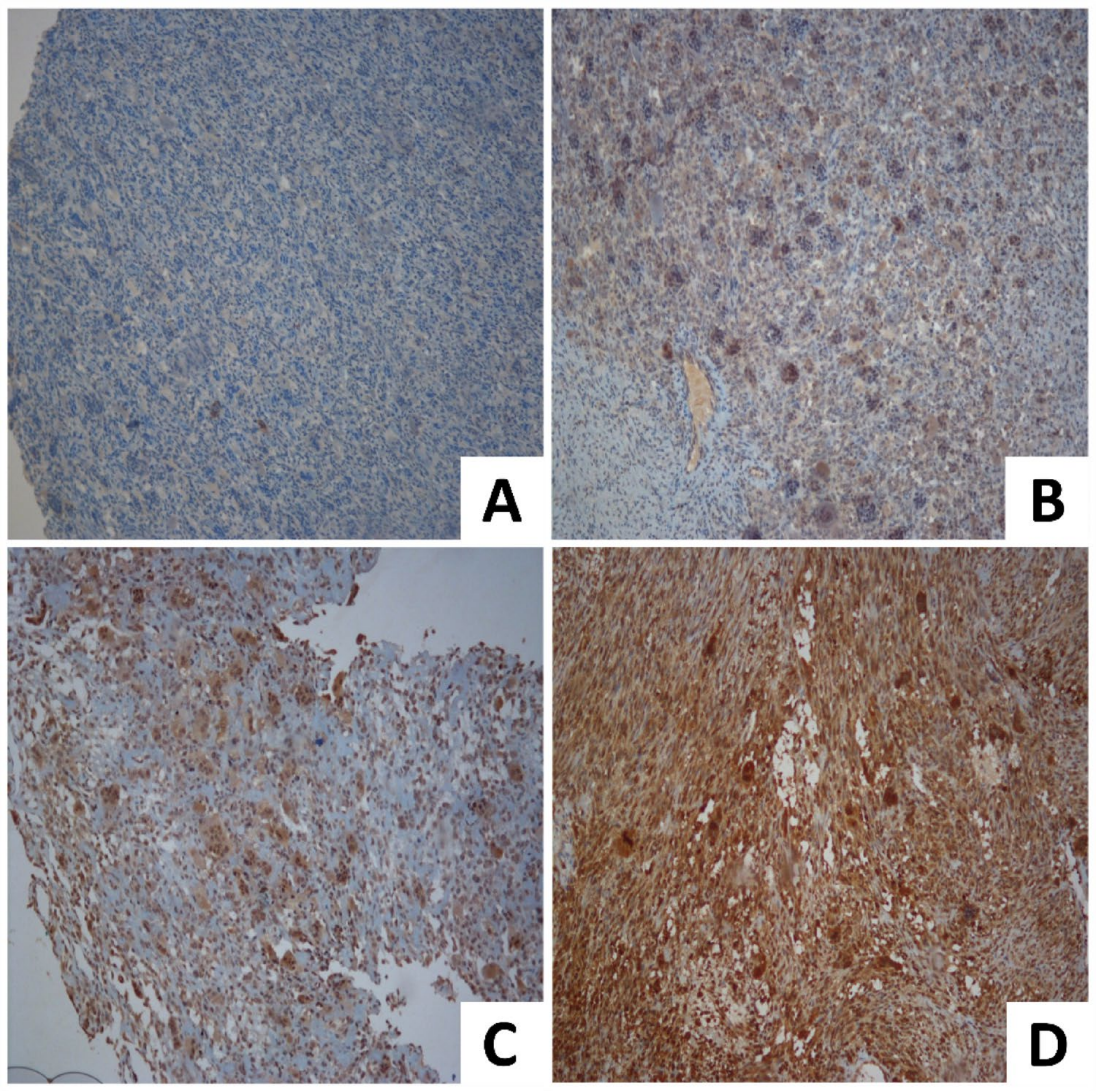
HtrA1 Immunostaining: Grade 1 staining in both giant cells and mononuclear cells (**A**). Giant cells showing Grade 3 staining, while mononuclear cells show Grade 2 staining (**B**). Giant cells showing Grade 4 staining, and mononuclear cells showing Grade 3 staining (**C**). Grade 5 staining in both giant cells and mononuclear cells (**D**)

We assessed HtrA1 expression in mononuclear and giant cells using immunohistochemistry with a specific antibody (Fig. 2). HtrA1 immunostaining was evaluated by two pathologists experienced in musculoskeletal pathology, and final staining grades were determined by consensus. Staining intensity was scored using a predefined five-tiered semi-quantitative system based on the percentage of positive cells, with Grade 1 representing negative staining, Grade 2 indicating less than 25% positive cells, Grade 3 indicating 26–50%, Grade 4 indicating 51–75%, and Grade 5 indicating 76–100% positive cells (Fig. 3).

For categorical analysis, Grade 5 staining was defined as high expression, as it represented diffuse HtrA1 positivity in more than 75% of the evaluated cells. Grades 1–4 were grouped as low expression, representing absent to non-diffuse staining. Giant cell (GC) and mononuclear cell (MNC) staining were then each dichotomized according to these criteria. For statistical analyses, “High GC” and “High MNC” were used to indicate high HtrA1 expression in giant cells and mononuclear cells, respectively, whereas “Low GC” and “Low MNC” were used to indicate low HtrA1 expression in the corresponding cell populations.

### Statistical analysis

Data were analyzed using SPSS version 27 software. Continuous variables were assessed for normality using the Shapiro–Wilk test. Age was normally distributed and summarized as mean (range), whereas follow-up duration and time to recurrence were non-normally distributed and summarized as median (range). Categorical variables were summarized as frequencies and percentages. Fisher’s exact test was used to compare recurrence rates between HtrA1 expression groups and to evaluate the associations between recurrence and potential treatment-related confounders, including Campanacci grade, reconstruction method and local adjuvant use, because of the small sample size and low expected cell counts. Exploratory binary logistic regression was performed to estimate the association of High GC and High MNC expression with recurrence, and results were reported as odds ratios with p-values. Recurrence-free survival was evaluated using Kaplan–Meier analysis, and HtrA1 expression groups were compared using the log-rank test. Due to the limited number of recurrence events, multivariable adjustment was not performed. A p-value of less than 0.05 was considered statistically significant.

## Results

The patient cohort demonstrated a slight female predominance, with a female-to-male ratio of 1.4 and a mean age of 37.6 years (range, 18–56). The most common tumor sites were the distal femur and proximal tibia, accounting for 64.7% of cases. All patients underwent a Jamshidi biopsy prior to surgery. Based on the Campanacci staging system, 8 patients (23.5%) had Grade I disease (well-defined borders), 15 patients (44.1%) had Grade II disease (poorly defined borders within the bone), and 11 patients (32.4%) had Grade III disease (soft tissue extension). Regarding reconstruction after curettage, 22 patients underwent cementation and 12 underwent grafting. Local adjuvants were used in 20 patients, whereas 14 patients received no additional local adjuvant treatment. The median follow-up duration was 33 months (range, 24–141 months). The overall recurrence rate was 38.2%. These demographic and clinical characteristics of the patient cohort are summarized in Table 1. HtrA1 immunostaining was evaluated separately in giant cells and mononuclear cells, and the distribution of staining grades is summarized in Table 2.

**Table 1:** Demographic and clinical characteristics of the patient cohort

Variable		Value
Age, years, mean (range)		37.6 (18–56)
Sex, n (%)		
Female		20 (58.8%)
Male		14 (41.2%)
Tumor location, n (%)		
Distal femur		13 (38.2%)
Proximal tibia		9 (26.5%)
Distal radius		3 (8.8%)
Proximal humerus		2 (5.9%)
Proximal femur		2 (5.9%)
Distal tibia		1 (2.9%)
Proximal fibula		1 (2.9%)
Acetabulum		1 (2.9%)
Metacarpal		1 (2.9%)
Sacrum		1 (2.9%)
Campanacci grade, n (%)		
Grade I		8 (23.5%)
Grade II		15 (44.1%)
Grade III		11 (32.4%)
Surgical treatment, n (%)		
Curettage with cementation		22 (64.7%)
Curettage with grafting		12 (35.3%)
Follow-up duration, months, median (range)		33 (24–141)
Recurrence, n (%)		13 (38.2%)
Time to recurrence, months, median (range)		9 (1.5–47)

The associations of Campanacci grade, reconstruction method and local adjuvant use with recurrence were explored as potential treatment-related confounders. Recurrence was observed in 3 of 8 patients (37.5%) with Campanacci Grade I disease, 4 of 15 patients (26.7%) with Grade II disease, and 6 of 11 patients (54.5%) with Grade III disease. Regarding reconstruction method, recurrence occurred in 7 of 22 patients (31.8%) treated with cementation and in 6 of 12 patients (50.0%) treated with grafting. Recurrence was also observed in 7 of 20 patients (35.0%) treated with local adjuvants and in 6 of 14 patients (42.9%) without local adjuvant treatment. None of these variables showed a statistically significant association with recurrence (*p* = 0.352, 0.462, and 0.728, respectively). HtrA1 staining grades showed a mean of 3.76 and a median of 4 (range, 1–5) in giant cells, and a mean of 3.12 and a median of 3 (range, 1–5) in mononuclear cells. For categorical analysis, Grade 5 staining was considered high expression, whereas Grades 1–4 were considered low expression. In exploratory binary logistic regression, High GC expression showed a non-significant trend toward increased recurrence risk (OR = 5.75, *p* = 0.067), whereas High MNC expression showed a non-significant trend toward lower recurrence risk (OR = 0.15, *p* = 0.094). Based on these opposing directional trends, GC and MNC expression patterns were evaluated together by categorizing patients into four groups: High GC / High MNC, High GC / Low MNC, Low GC / High MNC, and Low GC / Low MNC. The groups and their corresponding recurrence rates are summarized in Table 3. Recurrence rates were higher in the High GC / Low MNC group compared with the Low GC / Low MNC group and all other patients combined; however, these differences did not reach statistical significance (*p* = 0.095 and *p* = 0.079, respectively). Among patients who developed recurrence, the median time to recurrence was 9.0 months (range, 1.5–47 months). Kaplan–Meier analysis showed that the High GC / Low MNC group had the shortest recurrence-free survival, although the overall difference among the four HtrA1 expression groups was not statistically significant (*p* = 0.233). Compared with all other groups combined, the High GC / Low MNC group showed a borderline but non-significant trend toward shorter recurrence-free survival (*p* = 0.052). The results of the statistical analyses are detailed in Table 4.

**Table 2:** Distribution of HtrA1 immunostaining grades according to cell type

Cell type	Staining Grade	*n* (%)
Mononuclear cells	Grade 1	4 (11.8%)
	Grade 2	12 (35.3%)
	Grade 3	2 (5.9%)
	Grade 4	8 (23.5%)
	Grade 5	8 (23.5%)
Giant cells	Grade 1	2 (5.9%)
	Grade 2	5 (14.7%)
	Grade 3	6 (17.6%)
	Grade 4	7 (20.6%)
	Grade 5	14 (41.2%)

**Table 3:** Staining groups and recurrence rates

Group	Number of cases	Recurrence (%)
High GC / High MNC	7	2 (28.57)
High GC / Low MNC	7	5 (71.43)
Low GC / High MNC	1	0 (0.00)
Low GC / Low MNC	19	6 (31.58)

GC, giant cell; MNC, mononuclear cell

**Table 4:** Statistical analyses of recurrence and recurrence-free survival among HtrA1 expression groups

Comparison Between Groups	*p*-value*	*p*-value†
High GC / High MNC vs. High GC / Low MNC	0.286	0.243
High GC / High MNC vs. Low GC / High MNC	1.0	0.578
High GC / High MNC vs. Low GC / Low MNC	1.0	0.943
High GC / Low MNC vs. Low GC / High MNC	0.375	0.193
High GC / Low MNC vs. Low GC / Low MNC	0.095	0.080
Low GC / High MNC vs. Low GC / Low MNC	1.0	0.423
High GC / Low MNC vs. the others	0.079	0.052

GC, giant cell; MNC, mononuclear cell. *Fisher’s exact test; †Log-rank test

## Discussion

Our study represents the first investigation of HtrA1 expression in GCTB, evaluating 34 cases by immunohistochemical analysis. We explored HtrA1 expression patterns in giant cells and mononuclear cells to assess their potential association with recurrence. When the cohort was categorized into four groups according to HtrA1 expression patterns, the High GC / Low MNC group showed the highest recurrence rate, at 71.43%, although this finding did not reach statistical significance. Survival analysis showed a similar non-significant trend, with the High GC / Low MNC group demonstrating the shortest recurrence-free survival. However, these findings should be interpreted as exploratory because of the small sample size and limited number of events. Clinical and treatment-related factors, including Campanacci grade, reconstruction method, and local adjuvant use, may influence local recurrence after curettage for GCTB. In the present cohort, none of these factors was significantly associated with recurrence. These findings suggest that the observed trend between HtrA1 expression patterns and recurrence was unlikely to be solely explained by differences in these treatment-related variables. However, given the limited sample size, residual confounding cannot be excluded.

Although no statistically significant differences were detected, the High GC / Low MNC pattern showed the strongest association with recurrence across both recurrence-rate and recurrence-free survival analyses. This pattern may represent a subgroup with a higher recurrence tendency; however, it remains an exploratory observation and should not be interpreted as confirmatory. No recurrence was observed in the Low GC / High MNC group, but this subgroup included only one patient, precluding meaningful interpretation. Therefore, these findings should be considered hypothesis-generating and require validation in larger cohorts.

Genetic research on GCTB was popularized over the past two decades. The recent discovery of the H3.F3A p.G34W mutation in GCTB offers a highly sensitive and specific diagnostic marker [13, 14]. While this mutation aids in GCTB identification, its utility in predicting prognosis remains unclear. Other proposed markers like Ki67, P53, cyclin D1, and β-catenin have shown limited reliability in predicting GCTB behavior [15]. Given the well-established tumor suppressor role of HtrA1 in various cancers and its diverse expression patterns in GCTB, it holds promise as a potential prognostic marker [16, 17].

Disruption of the extracellular matrix is one of the initial steps in tumor invasion, allowing the tumor to infiltrate surrounding soft tissues. Previous studies revealed the synthesis of extracellular matrix metalloproteinase inducers (EMMPRIN) in GCTB. This locally aggressive benign tumor exhibits strong EMMPRIN staining on the surface of multinucleated giant cells, with weaker staining observed in mononuclear cells [18]. The order of microscopic events begins with activation of NF-KB ligand by mononuclear stromal cell, followed by increased recruitment of multinuclear giant cell, increased expression of EMMPRIN, activation of matrix metalloproteinase, and degradation of the extracellular matrix.

The involvement of HtrA1 in various cellular processes relevant to GCTB development, including TGF-β signaling and bone remodeling, suggests its potential mechanistic role in the disease [12]. In osteoporosis, upregulation of HtrA1 was also observed in osteoporotic animal models related to the overactivation of the NF-ĸB pathway [19]. Recombinant HtrA1 protein has been shown to cause deterioration of cartilage structure in end plates and knee joints by increasing the release of MMP and ADAMTS5 molecules, as well as exerting a detrimental effect on type II collagen in ovariectomized rats. These findings have been supported by similar studies, which further elucidate the mechanism underlying this process [20, 21]. These studies showed a strong relationship between HtrA1 and the bone and extracellular matrix microstructure, similar to its role in osteoporosis. Additionally, HtrA3 downregulation has been observed in ovarian and endometrial cancers [22]. Given its structural homology to HtrA1, HtrA3 might also have a role in the same manner. Supporting this, another study identified a role for HtrA3 in osteosarcoma, where its downregulation was linked to a specific pathway formation in osteosarcoma [23]. Although our findings did not establish a definitive association between HtrA1 expression and the risk of local recurrence, they suggest that differential HtrA1 expression between giant cells and mononuclear cells may have prognostic relevance. The biological role of HtrA1 in GCTB may be complex and context-dependent. On one hand, HtrA1 may contribute to local tumor invasion through extracellular matrix remodeling and degradation; on the other hand, its previously described tumor-suppressive functions suggest that its biological effect may depend on cellular context and disease stage. The observed positive HtrA1 staining in both mononuclear and multinucleated giant cells, with a tendency toward higher expression in giant cells, provides initial insight into its distribution within the GCTB microenvironment. Further well-designed immunohistochemical and molecular studies are required to validate these preliminary findings and clarify the biological and prognostic role of HtrA1 in GCTB management.

This study acknowledges several limitations, including its retrospective design, relatively small sample size, and potential selection bias. The limited sample size and small number of recurrence events precluded multivariable adjustment for reconstruction method and local adjuvant use. Additionally, the study did not investigate the specific mechanisms underlying HtrA1 expression in GCTB and its possible role in tumor progression. Further studies should focus on expanding the sample size, particularly in underrepresented groups like Low GC / High MNC, and incorporate longitudinal data to validate these preliminary findings and refine the predictive value of HtrA1 expression patterns.

In conclusion, this study identified non-significant trends suggesting that differential HtrA1 expression in giant cells and mononuclear cells may be associated with recurrence in GCTB. Although conclusive evidence could not be established, these preliminary findings highlight the need for further studies to clarify the biological and potential prognostic relevance of HtrA1 expression patterns in GCTB.

## Data Availability

The study data will be available upon request to the corresponding author.
